# ShoeCase: A data set of mock crime scene footwear impressions

**DOI:** 10.1016/j.dib.2023.109546

**Published:** 2023-09-07

**Authors:** Abigail Tibben, Megan McGuire, Stacy Renfro, Alicia Carriquiry

**Affiliations:** Iowa State University: 3410 Beardshear Hall, Ames, IA 50011 CSAFE: 195 Durham Center 613 Morrill Road Ames, IA 50011, USA

**Keywords:** Footwear impressions, Forensics, Outsole impressions, Mock crime scene evidence, Outsole imprints dataset, Shoeprint evidence, Shoeprint forensics

## Abstract

This project's main objective is to create an open-source database containing a sizeable number of high-quality images of shoe impressions. The Center for Statistics and Applications in Forensic Evidence (CSAFE) team collected images that represented those found at crime scenes and constructed a database that is publicly available to forensic science and research communities. The database includes images obtained from mixed impression types: full blood impression, partial blood impression, and dust impression. The impressions are made on different flooring (vinyl and tile), and captured via various lift techniques: gel lifts and handiprints (exemplar prints with high definition using graphite powder and clear sticky vinyl, backed by a white sheet), and saved in multiple digital file types (TIF, XMP, CR3, and JPG). Our data were collected to ensure reproducibility, using simple but well-described protocols and easily accessible materials.

The complete dataset includes 936 unique shoeprint images saved in 3,275 digital files.

Data were collected by trained volunteers making shoe impressions on flooring with the two mediums: spatter blood and graphite powder. To make an impression, volunteers wearing a shoe stepped into the material and then walked on the flooring. A separate “lighter” step was taken to create a partial print for the blood prints. The blood prints were brought to the photography station, where researchers labeled and photographed them. Graphite prints were covered with a gel lifter before being moved to the photography room. There, the researchers removed the lift, labeled, and then photographed them.

Our data will be of significant use to researchers, examiners, and anyone who could benefit from using a large dataset like this. Footwear datasets are often difficult to find, especially ones that resemble crime scenes, so our data can help fill that gap.

Specifications Table

**Table:** 

Subject	Criminology
Specific subject area	Mock crime scene footwear impressions for forensic research and analysis
Type of data	Table Image
How the data were acquired	The prints were made using fingerprint powder and gel lifts, or spatter blood, using two types of shoes: Nike and Adidas. After print creation, they were photographed. The blood prints were photographed with a camera, tripod, flash attachment, light bouncer, and an angle scale. The gel lift images were taken with the same camera and angle scale but with a different tripod. Lighting was provided using a modified RC car to perform a photography technique called “light painting” for even lighting. After the Raw images were collected, Photoshop was used to crop and perspective warp the TIF files.
Data format	RAW (CR3) JPG TIF (cropped/fixed images) XMP
Description of data collection	The data were created and collected by making shoeprints on a substrate. Then, the prints were lifted with a gel lifter, or the print on the substrate was directly photographed, and the images were cropped and perspective warped as detailed in the protocol. The images are organized according to the substrate, shoe type, print medium, and print type.
Data source location	Center for Statistics and Applications in Forensic Evidence 195 Durham Center 613 Morrill Road Iowa State University Ames, Iowa 50011 USA
Data accessibility	Repository name: Iowa State University (ISU)Data share Data identification number: doi.org/10.25380/iastate.22814438.v1 Direct URL to data: https://doi.org/10.25380/iastate.22814438.v1

## 1 Value of the Data

• These data are useful for creating comparison algorithms, providing objective statistical backing to cases involving shoe impressions. They can also be useful for training in forensic fields and fill the data gap for shoe mark evidence.
• Forensic practitioners can benefit from the data by using them for training and research. Statistical researchers can also benefit from the data as tests for comparison algorithms. With the development of objective comparison algorithms, prosecutors, lawyers, and other criminal justice officials will benefit from having objective and statistical evidence to make stronger cases.
• These data can be used to advance statistical forensic research by supporting testing and development of comparison algorithms for use by forensic practitioners. They can also be used as examples for practitioner training, teaching, and practice. The protocols will be used for further database expansion and are critical in developing better mock crime scene images for the database.

## 2 Objective

One of the fundamental problems in forensic science is the need of more publicly available data, especially data representative of real casework. The Organization of Scientific Area Committees for Forensic Science (OSAC) has identified key places where forensic research needs improvement, and pattern analysis is one of them. Comparative algorithms that quantify the similarity between two impressions provide an objective way to statistically compare footwear evidence, but they cannot be developed or tested rigorously without data. In this light, lack of data remains a major barrier to developing quantitative methods for forensic footwear analysis. This project's objective is to create a large database of footwear outsole impressions representative of casework. By including a variety of media, substrates, and capturing techniques, each impression was recorded multiple times to enable the estimation of measurement error. Because only two shoe models were used in this study, these images can be used to test whether algorithms can detect close non-matches, the most challenging type of comparison made by examiners. Since the database is in the public domain, these images can be used beyond algorithm creation for forensic training, research, and examination. CSAFE intends to expand this initial dataset with more shoe brands, sole types, and sizes to more accurately represent populations of shoes.

## 3 Data Description

Two Excel sheets summarize the content of the databases and will be described in further detail below. The “current_protocol” PDF file also contains details on how the prints were made and how the prints were photographed and processed, as well as technical information on the data.

There are **five folders** with data:

• Nike Prints (blood)
• Adidas Prints (blood)
• Adidas Gel lifts
• Nike Gel lifts
• handiprint images

Users can use the Handiprint_database Excel sheet and the File_database Excel sheet to search for specific types of prints. **The file names** (except for the handiprints) are created using these rules:

• Shoe number (a three-digit pair ID number)
• R or L (right or left shoe)
• Type of print: B or G (blood or graphite)
• F or P (full or partial – for blood prints only)
• Flooring type: T or V (tile or vinyl this section is used only with blood impressions, as all gel lifts are made on vinyl)
• CAPF - crop and perspective fix, CR - cropped only (only if they are edited)
• Date the photo was taken (e.g., 9-10-22)
  • Date the file was edited (e.g., 9-15-22) if applicable
• Initials of the person who took the photo
  • Initials of the person who edited the photo are added on when the photos are edited, in addition to the information of who took the image, when applicable.

The file for the print database has two pages, one for blood prints and one for gel lifts. For simplicity, we will be summing the row counts, as each row is a separate file.

• Number Of Variables/Columns: 12 on each page (same prompts for each print)
• Number Of Cases/Rows: 780 total, excluding the two rows with labels.

Variables in the File_database sheet:

**Table:** 

Name	Description	Values
Shoe #	The ID number unique to each pair of shoes, followed by L or R to denote which shoe is used.	A three-digit number followed by L or R
Base file name	The file's name in the folders can be used to search for specific prints. Edited images will share the base file with an additional date and initials at the end.	Refer to the file names section in the file list category. Example: 005LG02-04-23ATMM
Print type	Notes whether the print is full or partial. Full contains at least 75% of tread detail and partial contains ∼50% of tread detail.	The words “full” or “partial”
Medium	The material that was coated on the shoe sole to make a print.	The words “fingerprint powder” or “synthetic blood”
Flooring material	The substrate on which the print was created. Note: all gel lifts are created on vinyl.	The words “tile” or “vinyl”
Lifting technique	The material that was used to lift the print off the flooring. Note: used only for gel lifts.	The words “none” or “gel lifts”
Notes	Any information regarding the quality of the print, especially when cropped or edited.	The cell will be blank or contain notes such as “cropped past 300” to explain that the print goes beyond the normal cropping measures.
Manufacturer	The brand of the shoe	The brand “Nike” or “Adidas”
Style	The name of the style of shoe	The words “Zoom Winflo 4” or “Seeley”
Size (US)	The size of the shoe in US sizes	A numerical value between 6.5 and 11
Category	Type of shoe, such as a sneaker, boot, or heel	Listed as: “Sneaker”, “Formal”, “Boot”, and “Heel”
Type	Use of shoe, such as a running, dress, or casual shoe	The words “casual”, “work”, “dress”, or “running”

For the handiprint file:

• Number of Variables/Columns: 4
• Number of Cases/Rows: 156 (excluding label row 1)

Variables for the Handiprint_database sheet:

**Table:** 

Name	Description	Values
File name	The name of the file in the folders. The meaning of the names is inconsequential, and the other three categories will match the handiprint to each shoe.	Numerical and alphabetical title Example: 002054L_20180411_5_1_1_hanrahan_zwart_jekruse
L/R	Whether the shoe is a left or right shoe	Noted as “L” for left shoe and “R” for right shoe
ID #	The ID number unique to each pair of shoes	A numerical value from 1-160
Adidas/Nike	Brand of the shoe	Brand “Adidas” or “Nike”

## 4 Experimental Design, Materials, and Methods

### 4.1 Materials

Shoe prints obtained using graphite powder

• Fingerprint dusting graphite powder and brush
  ○ Evident brand, item #1026, Black Fingerprint Powder 2oz
• Gel lifters
  ○ Gel lifters brand, Footprint Lifters Black, 13 × 36cm, article # B-12000

or

  ○ Gel lifters brand, Footprint Lifters Black. 18 × 36cm, article # B-12500
• Printer paper
• Flooring material
  ○ vinyl
• 78 test shoe pairs
  ○ 39 pairs of Adidas Seeley's* (size range from 7-10)
  ○ 39 pairs of Nike Zoom Winflow 4s* (size range from 7-10)
• Garbage bags or tarp
• Lint roller
• Wipes to clean (e.g., Clorox)
• Paper towels (brand doesn't matter) for clean up
• Disposable gloves
• Labels for shoes in the image

Shoe prints made with spatter blood

• Spatter blood
  ○ *Evident* brand, Spatter Training Blood 8oz, SKU 3675
• Shallow tray/plate (plastic or metal, nothing fragile as it will be stepped on numerous times)
• Viva brand paper towels (for blood plate)
• Flooring material
  ○ Tile
  ○ vinyl
• 78 test shoes
  ○ 39 pairs of Adidas Seeley's* (size range from 7-10)
  ○ 39 pairs of Nike Zoom Winflow 4s* (size range from 7-10)
• Garbage bags or tarp
• Wipes to clean (e.g., Clorox)
• Paper towels (brand doesn't matter) for clean up
• Labels for shoes in the image
• Optional: disposable gloves

*Note: This dataset is made for testing algorithms to determine whether prints match each other. The expansive number of shoes (though only 2 types) are meant to test whether the algorithm can distinguish close non matches as well as more “obvious” differences such as size and style. Because these algorithms are being created and are in the early testing stages, having an expansive collection would make it difficult to determine what exactly is breaking the code. We have started out with 2 shoe types both for practicality (we already had a large quantity of both shoes) and because it would allow us to test algorithms on 2 different levels: distinguish between shoe brands (more “obvious”) and close non matches, which would be distinctions within the same shoe brand (RACs, size, etc.). CSAFE is currently expanding this dataset with more shoes, and we intend to make this dataset as large and robust as possible.

## 5 Protocols for the Collection of Shoeprints

Blood prints:

Using each shoe, we make two types of prints (full and partial). Each print is made on vinyl and tile. As a result, we obtained four prints using blood for each shoe in the test set.

1. Setup
  a. Put a bag or tarp down in the workspace to prevent carpet staining.
  b. Place flooring on the cover.
  c. Cut a Viva brand paper towel to fit on the tray, ensuring the towel reaches the edge but does not go over. Use one to two paper towel layers, so the towels soak up the blood and make a stamp pad to step on.
  d. Pour around four tablespoons of spatter blood onto the plate evenly and wait for it to be absorbed. Make sure that the paper towel is saturated but that there is no pooling.
  e. Place the plate on the floor next to the flooring on which the print will be made.
  f. Put on the shoe of choice and step on the plate, ensuring that the entire bottom of the sole comes into contact with the blood. For each new step print, recoat the shoe in the fake blood.
2. Full-step print
  a. Once the shoe outsole is coated, take a single step onto the flooring of choice using a natural gait.
  b. If the print does not come out well (e.g., the print includes pools of blood or shows a partial print), clean the flooring and try again. If the blood is too old, it can begin to gel; it looks like pink powder when it dries. If this happens, refresh the blood tray (you can try shaking the blood bottle or using a new bottle) and redo the print.
  c. Take the shoe off immediately and set it aside.
  d. Let the print dry fully to reduce glare on the image (around 5-8 minutes)
  e. When the print has dried, it can be photographed. The protocol for photographing blood prints is presented below.
3. Partial print
  a. Once the shoe outsole is coated, walk/run onto the flooring on the ball of your foot, how you would if you were running. You can also walk on your heel or toe and get a non-smudged half-print.
  b. Take the shoe off immediately and set it aside.
  c. Let the print dry fully to reduce glare on the image (around 5-8 minutes)
  d. When the print has dried, it can be photographed. The protocol for photographing blood prints is presented below.
4. Cleaning Process (after images have been captured)
  a. Use a wet wipe to clean spatter blood off the flooring surface.
  b. It is crucial that ALL red residue is removed from the flooring so the contrast of the print and flooring doesn't change from shoe to shoe.
  c. Follow with a dry paper towel to remove excess cleaning liquid.
  d. Return the shoes to the drawstring bag and put them in a place separate from the collection area so they are not mistakenly used again.

Graphite prints:

Note: obtaining prints using graphite powder is more labor-intensive and done more efficiently when two individuals participate in the process.

1. Setup:
  a. To begin, put garbage bags down over the floor to protect it from being stained by the graphite powder.
  b. Place vinyl flooring material down on the garbage bags.
  c. Using a brush, dust a small amount of graphite powder onto a sheet of paper.
  d. Before stepping onto the paper, tap off excessive powder back into the container; ideally, some dust will be visible on the paper.
  e. Put on pair of test shoes
  f. Roll the sole's entire surface in the powder to ensure full coverage from toe to heel. Rubbing the shoe back and forth across the paper can help disperse the powder across the sole of the shoe.
  g. The outsole should be black or almost black on the parts of the outsole that will make contact with the floor. If you end up with powder in the crevices between the pattern elements, Tap the shoe, allowing excess powder to fall onto the paper. Add more graphite powder to the paper if obtaining a uniform coating on the outsole pattern elements is difficult.
2. Preparing flooring material:
  a. Between each graphite impression, clean the vinyl flooring material, first with a cleansing wipe and then with a dry paper towel.
  b. Immediately before making an impression, use a lint roller on the flooring to remove all potential dust contamination.
3. Creating the print:
  a. Step in graphite powder to coat the shoe sole, then walk onto flooring material, leaving one footprint (normal gait).
  b. If the print has excessive amounts or clumps of dust, wipe off the print and retry. If there is excessive smearing or a partial print, try it again.
  c. Remove the shoe while still on the tarp, and take the shoe away from the immediate area where the impression is being lifted.
  d. After putting on gloves, peel off plastic from the gel lift and place it on top of flooring material to get an impression of the print, touching the gel as little as possible. In more detail:
    i. Put one short end of the gel lift onto the flooring surface first, then lower the rest over the print as you press the gel lift down. Push out air bubbles with your fingers and ensure the lift is fully secured to the flooring.
    ii. Let the gel lift rest on the flooring for 4 minutes.
    iii. Peel the gel lift off the flooring material carefully, trying to touch the black gel as little as possible.
4. Cleaning Process (after gel lift is created)
  a. Use a wet wipe to clean graphite powder off the flooring surface.
  b. Follow with a dry paper towel to remove excess cleaning liquid.
  c. Run a lint roller over the surface to remove any air contaminants or fibers from the paper towel.

### 5.1 Capturing images using photography

Materials needed for capturing images

• Camera(s) and flash attachments. The images in the database we have constructed were captured using:
  ○ Canon, T8I camera
  ○ 18-55mm kit lens
  ○ Flash attachment: Cannon Speedlite 600EX II-RT (used for blood prints only)
• Tripod(s):
  ○ Magnus Pv-3310 (for gel lifts only)
  ○ Abithid Horizontal Tripod (for blood prints)
• Angled scale(s)
• Plywood spray-painted in matte black
• Light reflecting panel:
  ○ Westcott 30” illuminator reflector 2 in 1 (For blood prints)
• External lighting (for gel lifts only)
• Rufus: Modified RC car (Sharper Image, MPN 693101760) with Craftsman 60 lumen (UPC: 040082800181, Model 73965) hands-free work light attached at 8” off the ground.
  ○ PVC piping fitted to create a circle with about a 102 cm radius. The print to be photographed will be place in the middle of this ring.

General camera preparations

• For blood prints:
  ○ Using a level, ensure that the camera is parallel to the print and that the scale in the image is a 90-degree angle.
• For both the blood and the graphite prints:
  ○ Ensure focus is equal across corners of the impression.
  ○ Ensure that the heel is in the corner of the scale and the angle scale goes along the top and right side of the print/ gel lift.
  ○ If needing to get closer to fill the frame more, move the tripod down as opposed to zooming in with the camera. Remember if you move the camera, make a note of changes, and once you begin taking images, do not change any aspect of the lighting camera or tripod without redoing any previously done images for use.

Camera setup (Blood prints)

• Ensure that the print is centered in the frame and align the top and bottom of the flooring on which the print was made with marks on the painted board.
• The gold (sunlight) side of the lighting reflector is used.
• Place the angle scale on the floor close to the print, but not over it. Ensure that the heel is at the angle of the scale. Place the correct label on the angle scale.
• Place the tripod and reflector so that they can capture the entire print:
  ○ Distance from the print to the lens of the camera: 77.5 cm
  ○ Distance between all three tripod legs: 61.5 cm
  ○ Length of the arm from the tripod attachment to the camera attachment clip: 23.5 cm
  ○ Length of all 3 legs: 95.5 cm
  ○ Light reflector distance from the print: 36 cm
  ○ Reflector distance from flash attachment: around 26 cm
  ○ Reflector angle: 90 degrees (perpendicular to the flooring)

Camera settings:

• Shutter speed: 1/4 of a second
• Aperture: 25
• ISO: 1600
• 35 mm focal length
• Auto-select every other option
• Flash attachment:
  • Set to ETTL
  • No zoom
  • Flash settings at –2
  • Bent to ‘45’ line

Camera setup (Gel lifts)

• Place the gel lift in the marked space in the center of the PVC pipe ring.
• Place the angle scale on the gel lift close to the print with the heel in the angle. Place the correct label on the angle scale.
• Ensure that the tripod is set up to cover the entire gel lift:
  ○ Length of front two tripod legs: 70 cm
  ○ Length of the back tripod leg: 71.5 cm
  ○ Distance from the center of the print to the lens: 26 cm
  ○ Lens angle: about 15 degrees. You do not want the lens to be parallel due to the camera flash and glare issues, so it should be spaced far enough back to where the whole lift can be captured, but the camera is not directly above the print.
  ○ Diameter of ring track: about 203 cm
  ○ Flashlight on the RC car should be about 20 cm off the ground

Camera settings:

• Shutter speed: 15 seconds
• Aperture: 25
• ISO: 3200
• 35 mm focal length
• Auto-select every other option

Troubleshooting the gel lift image process

• The gel lift should be immediately taken to a dark area where photos will be taken and ideally away from the area where the impressions are being made to avoid dust contamination.
  ○ The edges of the gel lift begin to curl after it is peeled off the floor, creating glare around the edges. Take a photograph quickly before this occurs.
  ○ The low-light environment in the photography area may keep the camera from operating when one presses the shutter. If this occurs, increase light by, e.g., turning on a cell phone flashlight, but turn it off as soon as the shutter is pressed. During the 15-second exposure time, the circular stage defined by the PVC piping is light-painted using a modified RC car with a Craftsman flashlight. The car circulates on dowel rods 20 cm above the ground, carrying the flashlight that illuminates the stage at a speed such that it gets to complete one full trip around.
    ■ It can be helpful to have two people in the image-taking area to help turn off and on the light and arrange the scale.
• The speed with which the RC light car travels around the stage must be carefully determined. If the car moves too fast, it can jump off the rails. When the car travels at a low speed, it does not complete one cycle around, and this can reduce the amount of light on the print. To maintain a reasonable speed and to keep the car on the track, move the RC car in short bursts during the entire exposure period.
• If glare from the camera flashes in the image, move the tripod back an inch or so and angle the camera so the gel lift is in the frame.

### 5.2 Guidelines to name files

Shoeprint labels (included in the image itself)

• Shoe number
• R or L (right or left shoe)
• F or P (full or partial – for blood prints only)
• Put a label in the rectangle on the scale itself oriented so it can be read on the image

File naming (pre- and post-edits)

• Shoe number
• R or L (right or left shoe)
• Type of print: B or G (blood or graphite)
• F or P (full or partial – for blood prints only)
• Flooring type: T or V (tile or vinyl for blood prints only)
• Date the photo was taken (e.g., 9-10-22)
  ○ Date the photo was edited (e.g., 9-15-22)
• Initials of the person who took the photo
  ○ Initials of the person who edited the photo will be added upon editing, as well as the date

### 5.3 Editing images before inclusion in the database

Orientation and cropping

• We standardized the placement of the forensic angle scale so that in every image, the scale appears on the top right corner. The heel of the shoe is in the same corner and the toe area points to the left.
• Images were cropped at the 300 and the 145 marks on the scale, but if the print extended beyond that, we cropped to the edge of the print and made a note of it in the spreadsheet, as part of the metadata associated with each image.

Perspective fixing (for angle distortion)

To adjust images for angle distortions, we carried out the following steps:

1. Upload the image in Photoshop and select *edit* and *perspective warp*.
2. Click on the image and drag the corner points to each of the three targets in the scale.
3. Move the last corner point to the bottom left corner while keeping the lines straight and at right angles with corners above and to the right.
4. Click on the warp button and then click the # sign. This step aligns the image and fixes perspective issues.
5. Click the checkmark to apply the changes before saving the edited image.

## Ethics Statements

The study does not involve experiments on humans or animals.

## CRediT authorship contribution statement

**Abigail Tibben:** Methodology, Investigation, Data curation, Visualization, Writing – original draft. **Megan McGuire:** Validation, Investigation, Data curation, Visualization. **Stacy Renfro:** Conceptualization, Supervision. **Alicia Carriquiry:** Resources, Project administration, Funding acquisition, Writing – review & editing.

## Data Availability

ShoeCase: A data set of mock crime scene footwear impressions (Original data) (ISU Datashare)
